# A State-Level Examination into Structural Racism and Racialized Disparities in Sexually Transmitted Infections

**DOI:** 10.1007/s40980-025-00136-4

**Published:** 2025-02-10

**Authors:** Megan Evans, Lauren Newmyer

**Affiliations:** 1 Max Planck Institute for Demographic Research, Konrad-Zuse-Str. 1, 18057 Rostock, Germany; 2 Max Planck-University of Helsinki Center for Social Inequalities in Population Health, Rostock, Germany; 3 Department of Sociology, Bowling Green State University, Bowling Green, OH USA

**Keywords:** Sexually transmitted infections, Structural racism, Racial and ethnic disparities, United States

## Abstract

The population health literature recognizes structural racism as a fundamental determinant of racialized health disparities. However, the role of structural racism in the continued persistence of racialized disparities in sexually transmitted infections (STIs) has not been investigated despite Black Americans’ disproportionate experience of STIs in comparison to White Americans. Past research has largely investigated individual racial/ethnic identity as an individual-level factor predictive of STIs, failing to engage with the multitude of racially structured contexts which likely shape STI rates. This study combines multiple datasets, including data from the Centers for Disease Control and Prevention, the American Community Survey, and the Current Population Survey, to conduct a state-level analysis investigating the role of structural racism in contributing to Black–White racialized disparities in STIs between 2010 and 2020. Random effects spatial autoregressive models suggest that structural racism contributes to Black–White racialized disparities in STIs. This research contributes to literatures on structural racism and population health by better understanding how racialized state-level institutions shape the contraction of infections. The results have important implications for understanding states as institutional actors relevant for patterns of population health and the geography of racism.

## Introduction

Sexually transmitted infections (STIs) are currently at record high levels in the United States (U.S.). The CDC estimates that one out of five people experienced an STI in 2018, costing the healthcare system approximately $16 billion in treatment and management (Centers for Disease Control & Prevention, 2021). These infections impose not only an onerous financial cost on the nation, but also inflict consequential health circumstances for the individuals who contract them such as repeat infections, long-term health issues, or infertility (Newton & McCabe, 2008; Tolnay, 1989; Trottier & Franco, 2006; Ward & Rönn, 2010). Underlying the increasing STI rate is a strong and persistent racial patterning of STI exposure, contraction, and treatment (Adimora & Schoenbach, 2005; Adimora et al., 2006; Harling et al., 2014; Leichliter et al., 2020, 2022). Racialized inequalities in STIs are pervasive between Black and White Americans, with Black Americans having a higher likelihood of contracting an STI in comparison to White Americans (Leichliter et al., 2022).

Past research documenting these disparities has largely investigated the role of individual-level factors such as sexual behavior, contraceptive access, and healthcare access in driving the disparate patterns (Adimora & Schoenbach, 2005; Harawa et al., 2011). However, an individualistic approach fails to engage with and feature the core role structural racism performs in creating and maintaining health disparities. When macro-level racialized inequalities are unaddressed, individual-level health outcomes are unlikely to change (Link & Phelan, 1995). A growing body of literature highlights the important contribution of structural racism for racialized disparities in health (Brown & Homan, 2022a, 2022b; Brown et al., 2022, 2023; Hardeman et al., 2022; Homan et al., 2021; Hummer, 2023). However, scholars have yet to empirically examine the relationship between structural racism and sexual health.

Structural racism likely plays an important role in creating and maintaining racialized disparities in STIs. Structural racism is multi-sectoral and systemic, embedded across multiple institutional domains that shape an individual’s risk of contracting an STI, from the education and political system to the housing, economic, and criminal-legal system. While exposure to structural racism within each domain will likely drive racially disparate STI patterns through multiple pathways such as shaping dating opportunities, access to sexual health care, and education around sexual health, the amalgamation of these inequalities, in addition to the interconnected nature of the domains suggests that exposure to structural racism shapes racialized inequalities in sexual health. The geographic location an individual is embedded in jointly shapes their exposure to structural racism and their risk of STIs (Ellen et al., 2004; Jennings et al., 2005, 2010, 2012; Newmyer et al., 2022). Exposure to structural racism occurs at multiple spatial scales. This study builds on the literature identifying states as important geographic contexts and institutional actors that shape disparate demographic and population health outcomes (Brown et al., 2022; Montez & Zajacova, 2013; Montez et al., 2019).

Our study investigates the geography of structural racism with a particular focus on the contraction of STIs. We seek to understand the foundational relationship between exposure to structural racism and Black–White racialized disparities in STI rates at the state-level. Although a comprehensive analysis of mechanisms is out of the scope of this study, we also investigate two measures reflecting the social relations of Black and White residents in a state via the Black–White interracial marriage rates as well as anti-Black prejudice reflected through Google Trends searches using the N* word. We follow recent work by Brown and colleagues (2022) that measures structural racism as a latent construct scale to better represent its multifaceted, interconnected, and institutional nature. We combine multiple data sources, which include the Centers for Disease Control and Prevention’s reports of 2010–2020 STI counts of chlamydia, gonorrhea, and syphilis by state, the American Community Survey, and the Current Population Survey, to provide an investigation into the structural determinants of STIs over the past decade. We employ random-effects spatial autoregressive models to examine the association between state-level structural racism and disparate STI patterns of Black and White Americans. This research contributes to literatures on structural racism and population health by demonstrating the importance of states as institutional actors and geographic contexts shaping racialized inequality in the contraction of sexually transmitted infections.

## Background

### Structural Racism and Population Health

Across most domains of population health, Black Americans are disadvantaged in comparison to White Americans, dying earlier and having a higher prevalence of comorbidities and disability (Geruso, 2012; Hummer, 2023). After the declines in life expectancy attributed to COVID-19, life expectancy at birth for Black Americans is a striking 5.8 years shorter than White Americans (Arias & Xi, 2022; Hummer, 2023; Woolf et al., 2021). The broader population health literature identifies structural racism as a fundamental determinant of persistent racialized health disparities (Brown & Homan, 2022a, 2022b; Brown et al., 2023; Hummer, 2023). Moving beyond an essentialized, biological notion of race and ethnicity, demographers and population health scholars recognize that race and ethnicity are sociohistorical constructs representative of exploitation and oppression (Bonilla-Silva, 1997; Martinez et al., 2023; Ray, 2022; Williams, 2012).

Racism is a social system varying across time and place, embedded in government and politics, the health care system, education, the economy, media and broader culture, and the criminal justice system (Bonilla-Silva, 1997, 2021; Hummer, 2023; Ray, 2022; Williams et al., 2019). Thus, structural racism is best understood as multi-sectoral and systemic (Bonilla-Silva, 1997, 2021), leading Black and White Americans to experience unequal exposure to risks as well as access to material and psychological resources (Brown & Homan, 2022a, 2022b). This unequal exposure can extend from institutional and governmental policies biased against Black Americans (Anderson, 2017; Korver-Glenn, 2018; Owens, 2020; Rothstein, 2017), to racialized inequalities in income, wealth, and access to health-promoting resources and opportunities (Shapiro, 2004; Sharkey, 2013), racially-biased medical professionals and medical teaching philosophies (Arnett et al., 2016; Gaskin et al., 2012; Hannah-Jones, 2021; Nelson, 2002; Vaughan Sarrazin et al., 2009), and actual physical and physiological violence against Black individuals (Roberts, 2016). Structural racism has been linked to racialized health disparities in infant mortality (Wallace et al., 2017), COVID-19 mortality (Brown et al., 2022; Siegel et al., 2022), maternal health (Crear-Perry et al., 2021), and myocardial infarction (Lukachko et al., 2014), among several other mental and physical health outcomes (Hummer, 2023; Williams et al., 2019). In sum, a wealth of literature demonstrates the link between structural racism and racialized disparities in population health.

Simultaneously, with the growing body of research linking structural racism to population health, there are continued efforts on understanding how to best conceptualize and measure structural racism (Brown & Homan, 2022a, 2022b; Homan et al., 2021; Hummer, 2023). Scholars are still in the early stages of the complex task of adequately measuring the multi-sectoral, systemic nature of structural racism. While some scholars have used multiple measures simultaneously to represent different institutional aspects of structural racism (e.g., Chantarat et al., 2022; Lukachko et al., 2014), more recent work by Brown and colleagues, among others, have created a single latent scale that represents structural racism across multiple domains (Brown & Homan, 2022a, 2022b; Brown et al., 2022; Hing et al., 2024). The multidimensional measure is able to better capture the systemic nature of structural racism than unidimensional measures which more accurately capture institutional racism of a single domain (Brown et al., 2022; Dean & Thorpe, 2022; Hardeman et al., 2022; Hing et al., 2024). Exposure to structural racism occurs at multiple geographic scales (Hing et al., 2024). In this study, we build on past work from Brown et al. (2022, 2023) by adapting their state-level measure of structural racism to investigate whether its relationship to population health extends to racialized disparities in sexual health. Their latent measure captures structural racism within five interconnected domains, including the domains of education, housing, economic, criminal-legal, and political, which shape health disparities individually as well as collectively.

### Structural Racism and Sexually Transmitted Infections

Research investigating racialized disparities in STIs at the individual level have highlighted the role of segregated sexual networks (Bearman et al., 2004; Laumann & Youm, 1999; Liljeros et al., 2003), inequities in access to sexual health care (Arnett et al., 2016; Kirby et al., 2006), sexual health care utilization behaviors (Gilmore & Somerville, 1994; Lichtenstein, 2008; Lichtenstein et al., 2005), and inequities in quality of sexual health care received (Nelson, 2002). At the macro-level, however, it is exposure to structural racism which is the fundamental cause shaping each of these individual-level mechanisms (Link & Phelan, 1995; Williams & Collins, 2001). It is likely that exposure to structural racism within the five interconnected domains of education, housing, economic, political, and criminal-legal, individually, and collectively, shape individual level exposure to STIs and subsequently macro-level racialized disparities in STI rates. Thus, the population health literature requires an exploration into the fundamental relationship between exposure to structural racism and adverse sexual health outcomes.

In the interconnected domains of housing, education, and economics, it is well documented that racial segregation forces Black residents to disproportionately live in neighborhoods with higher rates of poverty and crime (Sharkey, 2013), which are farther away from adequate medical care (Arnett et al., 2016), and which are located in public school districts with inadequate resources and educational programs (Chetty et al., 2016; Owens, 2020). Educational inequalities can shape racialized disparities in STIs directly through lower quality school-based education on sexual health (Vivancos et al., 2013), which increases the risk of contracting STIs. Inequalities in economic and housing opportunities are associated with limited access to and affordability of healthcare, including sexual healthcare (Arnett et al., 2016; Kirby et al., 2006; Nelson, 2002). Past research demonstrates that Black residents living in disadvantaged neighborhoods have the highest rate of gonorrhea, compared to all other racial and ethnic groups (Springer et al., 2010). Within the criminal-legal sector, it is well documented that Black individuals have the highest incarceration rates in the United States (Pettit & Gutierrez, 2018). Higher rates of incarceration can lead to racialized patterns in STIs, as research documents high rates of STI transmission occur in carceral institutions (Nowotny et al., 2020). Scholars also document higher rates of STI transmission among the sexual partners of previously incarcerated individuals (Khan et al., 2011; Wiehe et al., 2015). Lastly, the continued disenfranchisement of Black voters leads to racialized disparities in voter turnout (Anderson, 2017). States play an important role in determining the sexual health education taught in public schools, access to healthcare through Planned Parenthood, and policy on the prevention of HIV (Adimora et al., 2014). Thus, structural racism within the political domain can shape policies that disadvantage Black Americans and contribute to racialized disparities in STIs. Taken together, there is likely a foundational relationship between exposure to structural racism, as represented as a systemic, interconnected, multi-dimensional measure, and racialized disparities in STIs, though it has yet to be tested.

While a full investigation into the mechanisms linking exposure to structural racism and racialized disparities in sexual health is out of the scope of this study, interracial social and sexual relations could influence unequal exposure to STIs. Racially segregated sexual networks are a key contributor to high rates of sexually transmitted infections among Black Americans (Adimora & Schoenbach, 2005; Liljeros et al., 2003). Physical and social racial segregation can lead to the racial segregation of sexual networks through social norms on dating (Bearman et al., 2004; Laumann & Youm, 1999; Liljeros et al., 2003). Anti-Black prejudice and racial animus can also lead to the mistreatment of and discrimination against Black residents in housing (Turner et al., 2014), job applications (Pager, 2003; Pager & Quillian, 2005), and availability and quality of health care (Nelson, 2002). Thus, this study also investigates the relationship between racialized disparities in STIs and the social and sexual relations of Black and White residents.

## Methods

### Data

We combine multiple datasets to investigate the relationship between structural racism and racialized disparities in state-level sexually transmitted infections. The Centers for Disease Control and Prevention (CDC) (2010–2020) provides data on the number of STI cases in each state by race, gender, age, and infection. Following guidance established by Brown and colleagues (Brown & Homan, 2022a, 2022b; Brown et al., 2022), we create a latent state-level structural racism scale combining multiple data sources including the Current Population Survey (CPS) and its Voting Supplement, American Community Survey (ACS), and the Uniform Crime Report (UCR). The CPS (2010 to 2020) provides us with information on racialized disparities in education, unemployment, poverty, and homeownership. The Voting Supplement from the 2008, 2012, 2016, and 2020 presidential elections provides us with data on racialized disparities in voter turnout. The UCR (2010 to 2020) provides data on racialized disparities in arrests. The ACS 5-year estimates (2008–2012, 2013–2017, and 2018–2022) at the census tract and state level provides us with population data which allows us to create an isolation index of segregation. We assign the 2008 to 2012 ACS to represent state-level dynamics in 2010, the 2013 to 2017 ACS to represent state-level dynamics in 2015, and the 2018 to 2022 ACS for 2020. We use linear interpolation to obtain values for the years in between (i.e., 2011 to 2014 and 2016 to 2019).

The ACS also provides us with population controls including the demographic and socioeconomic composition of the state. We use the micro-data from the ACS 1-year estimates to obtain household-level data on interracial marriages for each year between 2010 and 2020. Statistics of the Congressional Election provided by the Clerk of the U.S. House of Representatives provides information on state-level constituency returns for the elections of the U.S. presidency between 2008 and 2020, used to represent each state’s political leaning. Finally, we also incorporate Google Trends data (2010 and 2020) on Google searches using the N* word. Like other analyses of structural racism, we exclude 13 states from our analysis due to insufficient information on the states’ Black population (Brown et al., 2022).^1^ Though 13 states are excluded, 99% of the U.S. Black population lives within the states included in the final analytic sample. We also include the District of Columbia in our analysis which gives us a final sample size of 38 geographic locations with 11 years of observations (n = 418).

### Measures

#### Sexually Transmitted Infections

Our dependent variable is the state-level Black/White racialized disparity in rates of sexually transmitted infections for the population aged 44 and under. We measure STI rates by totaling the reported number of gonorrhea, chlamydia, and syphilis cases in each state for the population aged 44 and under divided by the respective populations (sourced from the Census) multiplied by 100,000. The Black/White disparity in STIs is created by taking the Black STI rate and dividing it over the White STI rate. Thus, our final measure represents racialized disparities in STI incidence rates for the population aged 44 and under.

#### Latent Measure of Structural Racism

The primary independent variable in our analysis is a latent scale of structural racism. We replicate Brown et al.’s (2022) state-level latent measure of structural racism to capture its multifaceted, interconnected, and institutional nature. Using an established measure allows us to build on this past research by further investigating the relationship between structural racism and population health. The latent construct uses seven measures to capture structural racism in five different domains: criminal-legal, education, economic, housing, and political. The measures include B/W ratio of arrest rates, W/B ratio of college completion rates, B/W ratio of unemployment rates, B/W ratio of poverty rates, W/B ratio of homeownership rates, W/B ratio of voting rates, and the White isolation index of racial residential segregation.^2^ All measures are calculated at the state level. Apart from arrest rates and the isolation index, these measures are sourced from the CPS. The arrest data is sourced from the UCR, and the ACS total population data is used to create rates, while the White isolation index is created using total population at the census tract level from ACS 5-year estimates data. Given 5-year estimates are necessary to create the isolation index and we assign the ACS 5-year estimates to 2010, 2015, and 2020, we only use these three years to create our structural racism measure and then use linear interpolation to obtain values of structural racism for the years in-between.

To replicate Brown et al.’s (2022) measure we use a confirmatory factor analysis (CFA) to capture the systemic and often unobserved nature of structural racism. Our CFA loads each structural racism dimension onto a single factor and allows for correlated errors based on both theoretical considerations and post-estimation assessments of the correlation matrix. We assess model fit using chi-square, RMSEA, BIC, CFI, TLI, and SRMR. Like Brown and colleagues (Brown et al., 2022) the best fit to the data comes from allowing W/B homeownership rates and W/B voting rates to be correlated as well as B/W arrest rates and B/W unemployment rates. Given the slightly different structure of our data, however, we obtain the best model fit by also allowing W/B college completion rates and W/B homeownership rates to be correlated as well as B/W homeownership rates and the isolation index, W/B voting rates and the isolation index, and B/W crime rates and the isolation index (*p* > χ^2^ = 0.212, RMSEA = 0.056, BIC = 959.288, CFI = 0.984, and SRMR = 0.045). We standardize our final structural racism measures for interpretability, where the mean is 0 and a standard deviation is 1.

#### Other State-Level Measures

The analyses control for the logged total population and a standardized Gini coefficient of income inequality. We account for the political leaning of the state with a dichotomous measure indicating whether the state voted for the republican candidate in the most recent U.S. presidential election (= 1). The analysis also investigates two measures representative of the state’s racialized social relations. The first measure is the percentage of Black–White interracial marriages. The prevalence of interracial marriages may be representative of more acceptance of Black Americans among White populations and may be indicative of less social and physical segregation between the Black and White population. The second measure is a standardized measure of Google searches using the N* word from Google Trends data. This measure represents an extreme expression of anti-Black prejudice and has been shown to be a strong predictor of racial segregation and voter turnout for Obama in 2008 (Rugh & Massey, 2014; Stephens-Davidowitz, 2014). Descriptive statistics are presented in Table 1.

**Table 1:** Descriptives Statistics, N = 418

	Mean/Prop	Min	Max
*STI Rates per 100,000*			
Total Rate	564.84	48.63	1383.41
Black Rate	2151.25	19.25	4600.88
White Rate	389.65	38.88	784.22
Black/White Ratio	5.94	.08	14.88
Latent Structural Racism	− .02	− 1.96	3.11
*Structural Racism Indicators*			
B/W Arrest Rate	3.13	.88	12.01
W/B College Completion Rate	1.63	.69	3.78
B/W Unemployment Rate	1.93	.21	7.23
B/W Poverty Rate	2.36	.79	6.22
W/B Homeownership Rate	1.68	1.05	4.38
W/B Voting Rate	1.28	.73	2.94
White Isolation Index	.90	.73	.97
*State-Level Covariates*			
Total Population, Logged	15.53	13.31	17.49
Gini Index of Income Inequality, Standardized	.08	− 1.97	3.37
Percent Black-White Interracial Marriages	.58	.24	1.86
Voted for a Republican Presidential Candidate	.50		
Anti-Black Prejudice, Google Searches using N* Word	.23	− 1.95	3.95

All STI rates represent the corresponding incident rate for the population aged 44 and younger. Mean/prop. statistics are averaged across the 2010–2020 period. The data covers the 37 continental states with a Black population of at least 50,000 and Washington D.C. This sample is consistent with studies of structural racism as it contains over 99% of the U.S. Black population

### Analytical Plan

Our analysis uses random-effects spatial autoregressive models. Spatial models allow us to account for the spatial dependence which exists in our state-level dependent variable, the Black/White ratio of rates of sexually transmitted infections. We incorporate a spatial error term for our dependent variable to model unaccounted for spatial autocorrelation present in the data. Preliminary spatial analyses indicate that a spatial error model is appropriate for our analysis. As our data is longitudinal, we use random effects models to investigate heterogeneity across U.S. state observations. This method essentially allows us to model unmeasured variables through the inclusion of a random intercept for each U.S. state in our data. We apply random effects models as we are interested in both between and within variation over time among states (Firebaugh et al., 2013).

## Results

### Racialized Trends in STIs

**Fig. 1:**
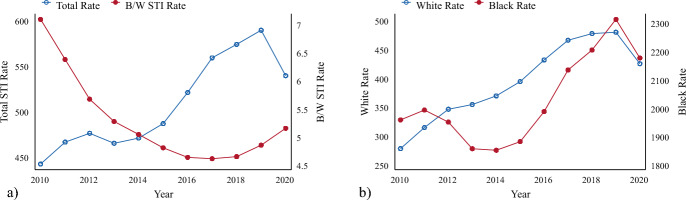
Rates of Sexually Transmitted Infections in the U.S. between 2010 and 2020 **a** Overall and **b** by Race. *Note*: All STI rates represent the incident rate of STIs for the respective population aged 44 and younger per 100,000. The data covers the 37 continental states with a Black population of at least 50,000 and Washington D.C. This sample is consistent with studies of structural racism as it contains over 99% of the U.S. Black population. In panel A, the total STI rate in blue, should be interpreted using the y-axis on the left; the B/W STI rate ratio in red should be interpreted using the y-axis on the right. In panel B, the blue line represents the White STI rate and should be interpreted using the y-axis on the left; the red line reflects the Black STI rate and should be interpreted using the y-axis on the right. (Color figure online)

Figure 1 presents the rates of sexually transmitted infections for the population under 45 in the U.S. between 2010 and 2020. Figure 1a shows the total STI rate in blue, which can be interpreted using the y-axis on the left, and the Black/White (B/W) STI rate ratio in red, which can be interpreted using the y-axis on the right. The figure demonstrates that the total STI rate rose by approximately 100 infections (per 100,000 individuals) between 2010 and 2020. While the rate of infected individuals declined slightly between 2019 and 2020, the CDC reports STIs have once again been on the rise since the COVID-19 pandemic began (Tanne, 2022). The figure also simultaneously demonstrates that as overall STI rates rose, the racialized disparity in STIs decreased during this period; however, the disparity rose once again between 2018 and 2020. Figure 1b helps clarify the concurrent rise in STIs and decline in racialized disparities. The blue line represents the White STI rate and can be interpreted using the y-axis on the left, while the red line reflects the Black STI rate and can be interpreted using the y-axis on the right. Figure 1b indicates both the Black and White STI rate increased between 2010 and 2020. The rate of increase, however, was much steeper among the White population in comparison to the low initial rate. The descriptives presented in Fig. 1 suggests that understanding structural drivers of STIs is warranted given the persistence of racialized disparities, increasing STI rates, and varying trends by race.

### Geography of Structural Racism and STIs

**Fig. 2:**
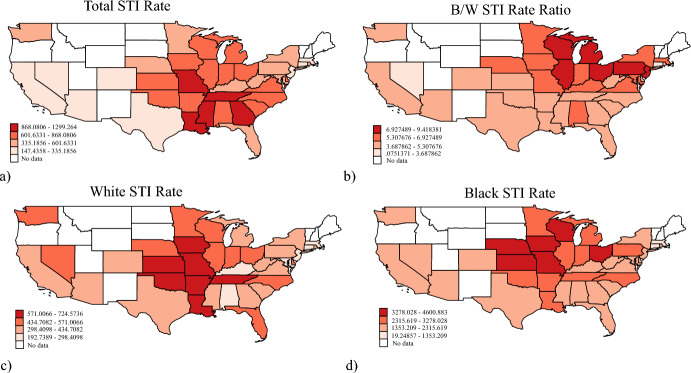
Maps of State-Level Variation in Rates of Sexually Transmitted Infections in the U.S. in 2020 by Standard Deviates **a** Total, **b** Black/White Ratio, **c** for the White Population, and **d** for the Black Population. *Note*: All STI rates represent the incident rate of STIs for the respective population aged 44 and younger per 100,000. The data covers the 37 continental states with a Black population of at least 50,000 and Washington D.C. This sample is consistent with studies of structural racism as it contains over 99% of the U.S. Black population

To demonstrate the spatial dependence present in our dependent variable, Fig. 2 presents maps visualizing the state-level distribution of the total STI rate, Black and White STI rate, and the Black/White STI rate ratio for the last year of observation in our study, 2020. The maps present STI rates by standard deviates and the states with the highest STI rates are colored deep red while the states in the lowest STI rates are colored light salmon. Figure 2 clearly showcases that STIs are highest in the Midwest and South, and lowest in the Northeast and West. Figure 2b also suggests that racialized disparities in STIs are highest in the Midwest, though they are also high in the Northeast despite the lower overall STI rate. A comparison of Fig. 2c, d indicates slight variation in which states have the highest and lowest STI rates for the Black and White populations.

### Spatial Autoregressive Models

Table 2 presents the results from the state-level random-effects spatial autoregressive models investigating the relationship between structural racism and racialized disparities in STIs between 2010 and 2020. Model 1 presents a bivariate investigation into the relationship between state-level latent structural racism and the B/W STI rate ratio. Model 2 includes controls for state-level sociodemographic and population dynamics, including the two measures of state-level racial social relations.

**Table 2:** Random-Effects Spatial Autoregressive Models Predicting State-Level Black/White STI Rate Ratio per 100,000 between 2010 and 2020, N = 418

	Model 1		Model 2	
	b	SE	b	SE
Latent Structural Racism	.65***	(.15)	.67***	(.15)
*State-Level Covariates*				
Total Population, Logged			− .09	(.29)
Gini Index of Income Inequality, Standardized			.12	(.25)
Percent Black-White Interracial Marriages			− 3.3***	(.67)
Voted for Republican Presidential Candidate			− .03	(.24)
Anti-Black Prejudice, Google Searches using N* Word			.32*	(.13)
Spatial Error Variance Parameter	.58***	(.04)	.54***	(.05)
Constant	5.9***	(.26)	9.3*	(4.7)
Pseudo R-Squared	0.077		0.112	
AIC	1436		1414	
BIC	1456		1455	

The Black/White STI Rate Ratio represent the racialized disparity in the incident rate of STIs for the population aged 44 and younger. The data covers the 37 continental states with a Black population of at least 50,000 and Washington D.C. This sample is consistent with studies of structural racism as it contains over 99% of the U.S. Black population. The Wald test of spatial autocorrelation is significant at *p* < .05. The spatial matrix for the spatial error term uses the Queen 1 criterion. *p < .05, **p < .01, ***p < .001

The results suggest that structural racism contributes to the continuation of racialized disparities in STIs in both the bivariate model and the adjusted model. In the bivariate model (model 1), a standard deviation increase in the latent structural racism scale is associated with an increase in a state’s B/W STI ratio of 0.65, which suggests that structural racism increases the predicted racialized disparity in STIs. This result persists even in the fully adjusted model (model 2), where we continue to see a significant and positive relationship between structural racism and the B/W STI ratio.

**Fig. 3:**
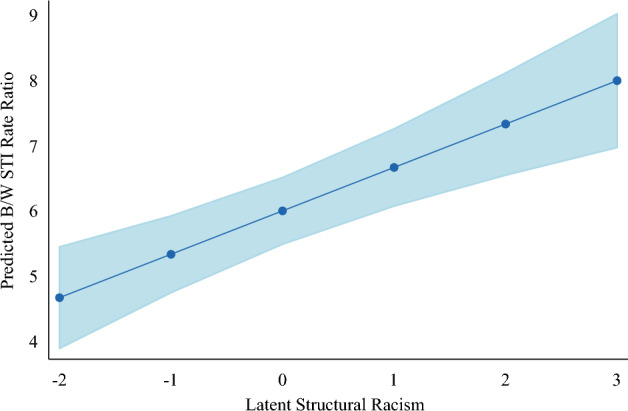
Latent Structural Racism and Predicted B/W STI Rate Ratio per 100,000. *Note*: The figure is produced using the fully specified random-effects spatial autoregressive model presented in Table 2. The latent structural racism measure is standardized to a mean of 0 and a one standard deviation of 1. The Black/White STI Rate Ratio represents the racialized disparity in the incident rate of STIs for the population aged 44 and younger. The data covers the 37 continental states with a Black population of at least 50,000 and Washington D.C. This sample is consistent with studies of structural racism as it contains over 99% of the U.S. Black population

Figure 3 presents the predicted B/W STI rate ratio by latent structural racism from the fully adjusted model (model 2) to aid in the interpretability of the results. States with values of latent structural racism one standard deviation below the mean have a predicted 5.3 Black residents who have contracted an STI for every 1 White resident per 100,000. In contrast, states with values of latent structural racism one standard deviation above the mean have a predicted 6.6 Black residents with an STI for every 1 White resident per 100,000, and states two standard deviations above the mean have a predicted 7.3 Black residents with an STI for every 1 White resident per 100,000. Overall, Fig. 3 helps illuminate that Black–White racialized disparities in exposure and contraction of STIs is quite high across all states. However, states that score the highest on the scale of latent structural racism have an additional 3 Black residents for every 100,000 who are unequally exposed to and contract an STI in comparison to states that score the lowest on the scale of latent structural racism.

In the fully adjusted model, we find no relationship between racialized disparities in STIs and the state’s total population, index of income inequality, or political leaning based on voting results for the most recent U.S. presidential election. However, we find both measures representing state-level racial social relations influence racial disparities in STI exposure and contraction. A one percentage point increase in the number of Black–White interracial marriages is associated with 3.3 fewer Black residents contracting an STI for every one White resident. We also find that states with higher anti-Black prejudice, reflected in Google searches using the N* word, is associated with higher racialized disparities in STI exposure and contraction. States with the least amount of anti-Black prejudice have a predicted 2 fewer Black residents that contract an STI in comparison to states with the most amount of anti-Black prejudice. Lastly, there is a significant spatial error term. A significant spatial error terms indicates that there continues to be unexplained spatial autocorrelation present in the data. This finding suggests that there are features not accounted for in the final model which make states that are geographically next to each other more similar to one another in their B/W STI rate disparity.

## Supplementary Analyses

We conduct multiple sensitivity analyses to assess the robustness of our results. Table 3 presents the correlation matrix of the measures included in our analysis and the structural racism measure, while Table 4 presents a set of models investigating the sensitivity of our findings to the inclusion of percent Black, percent in poverty, region, percent Black–White interracial marriage, and Google trends searches using the N* word. The table assesses how the bivariate relationship between structural racism and racialized disparities in STIs changes with the inclusion of each measure and presents the fully adjusted model with the inclusion/exclusion of the additional terms. Our main findings on the significance and magnitude of the relationship between structural racism and racialized disparities in STI remains robust to these alternate specifications.

As STI prevalence varies across age groups, Table 5 investigates the relationship between structural racism and racialized disparities in STIs among older adults, aged 45 and above. While we find a positive bivariate relationship between structural racism and the B/W STI rate ratio among the older population, this relationship disappears in the fully adjusted models. Rather, we find that increasing income inequality reflected in the Gini coefficient and that increasing the percentage of Black–White interracial marriages decreases the disparity in STIs among the population aged 45 and older. These results suggest that there might be distinct structural drivers of racialized disparities in STIs dependent on the population age, which aligns with past research finding fewer racialized differences in sexual behavior in older adulthood (Harawa et al., 2011).

We also investigate whether the relationship between structural racism and racialized disparities in sexual health varies by the type of STI contracted in Table 6. The results suggest that structural racism is associated with an increased racial disparity in exposure to and contraction of chlamydia and gonorrhea, but not syphilis. We also find that Black–White interracial marriages and anti-black prejudice influence racialized disparities in chlamydia rates, but not rates of gonorrhea or syphilis. Differences between structural drivers and STI types might be impacted by current infection trends of STIs, such as the rise in syphilis across the U.S. in all populations (Spicknall et al., 2021).

There are many scholars that have discussed the issue of scale when measuring residential segregation and its implications for understanding racialized inequalities (Lee et al., 2008; Lichter et al., 2015; Logan & Martinez, 2018). Our latent measure of structural racism incorporates segregation at the tract level, which can only capture micro patterns of segregation within a state. As a sensitivity test in Table 7, we also create a latent measure of structural racism that incorporates segregation at the tract level (micro) and county level (macro). The latent scale does not have as good model fit as the measure which only incorporates segregation at the tract level. Nonetheless, the results are robust when using the latent measure of structural racism that captures both tract and county level segregation. Additionally, Table 8 presents the results using the Black–White Dissimilarity index of segregation, rather than the White Isolation index, consistent with Brown and colleagues’ latent measure of structural racism. The results are robust using either measure of structural racism.

**Fig. 4:**
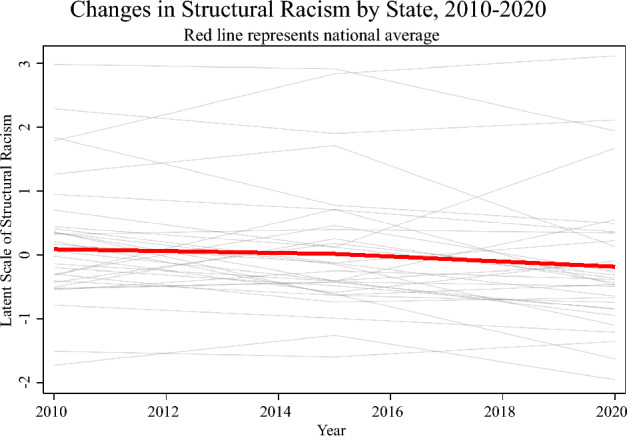
Changes in Latent Structural Racism by State between 2010 and 2020. Note: The latent structural racism measure is standardized to a mean of 0 and a one standard deviation of 1. The red line represents the national average in structural racism between 2010 and 2020. Each grey line represents an individual state. The data covers the 37 continental states with a Black population of at least 50,000 and Washington D.C. This sample is consistent with studies of structural racism as it contains over 99% of the U.S. Black population. (Color figure online)

Our final set of robustness checks uses the panel data to investigate variation in structural racism over time and the timing of exposure. On average, between 2010 and 2020 our measure of structural racism changes within a state by − 0.27 standard deviations, suggesting that over the last decade structural racism declines. Figure 4 graphs the variation in the latent scale of structural racism between 2010 and 2020. The red line represents the national average while each grey line represents an individual state over the period. The figure suggests that most states do not see major changes in their level of structural racism between 2010 and 2020. While we believe that presenting random effects models are more appropriate for our analysis than fixed effects models, Table 9 replicates the main results using fixed effects. The main findings are robust, with higher levels of structural racism increasing the predicted racialized disparity in STI rates, both within and between states. Table 10 replicates the main results using t − 1 lagged measures of the predictors. In the t − 1 lagged analysis we continue to find a strong positive association between structural racism and racialized disparities in STI exposure and contraction. In the lagged analysis, however, we no longer find a positive correlation between anti-Black prejudice and STIs.

## Discussion

Exposure to and contraction of STIs is of increasing concern for population health scholars as they continue to reach record levels (Leichliter et al., 2022; Tanne, 2022). As with many population health concerns in the United States, STI rates among Black Americans are five to eight times higher than rates among White Americans (Centers for Disease Control & Prevention, 2021; U.S. Department of Health & Human Services, 2020). Past research investigating racialized patterns of STIs has largely taken an individual approach, failing to engage with the complex institutional and population level dynamics which contribute to persistent racialized disparities (Bonilla-Silva, 2021; Brown & Homan, 2022a, 2022b; Brown et al., 2022; Evans & McDonald, 2023; Hummer, 2023). Our study advances work by Brown and colleagues (2022, 2023) by applying a theoretically informed measure of structural racism to highlight the foundational role state-level structural racism plays in racialized disparities in sexual health.

Findings indicate that structural racism is significantly related to unequal exposure to and contraction of STIs between Black and White individuals. At the state-level, latent structural racism is associated with an increase in the Black/White STI rate ratio. Thus, Black residents experience worse sexual health outcomes compared to White residents in states with more structural racism embedded within the housing, economic, education, political, and criminal-legal domains. These results are not surprising given the past research which connects components within each of these five domains individually to racialized disparities in STIs (Adimora et al., 2013; Bonney et al., 2012; Harling et al., 2013; Nowotny et al., 2020). Moreover, our results are consistent with the growing body of literature connecting structural racism to racialized disparities in population health (see Hing et al., 2024 for a recent scoping review). Our research further highlights the need to apply multi-dimensional measures of structural racism to capture its systemic and multidimensional nature. Racialized patterns in STIs are not explained by socioeconomic differences and individual-level variables, alone (Farley, 2006; Link & Phelan, 1995). While the link between racism and sexual health has been long been theorized on (Prather et al., 2016; Thompson et al., 2022), our study empirically demonstrates their foundational, state-level relationship.

Our study also highlights the importance of Black–White social relations for patterns of sexual health. High rates of interracial marriage are associated with decreased racialized disparities in Black/White STI rates. Our findings support past research identifying racially segregated sexual networks as a contributor to sexually transmitted infections among Black Americans (Adimora & Schoenbach, 2005; Liljeros et al., 2003), through their production of limited sexual partnering options in comparison to the typical sexual networks of White Americans (Bearman et al., 2004). The results have implications for understanding the relationship between assortative mating patterns, racialized sexual networks, and cross-race social relations. We also find that states with more anti-Black prejudice, represented through a larger number of Google searches using the N* word, have worse sexual health outcomes for Black residents than White residents. These results build on past research using Google Trends data to link anti-Black racial animus to higher levels of segregation (Rugh & Massey, 2014) and fewer votes for Obama in the 2008 presidential election (Stephens-Davidowitz, 2014). Our findings suggest that state-level racial animus also has important implications for shaping racialized patterns of population health, independent of structural racism as captured in our multidimensional scale.

Our study also contributes to the research identifying states as racializing institutional actors and geographic contexts which shape unequal population health outcomes (Brown et al., 2022; Bruch et al., 2019; Montez et al., 2019) likely through access to resources and opportunities, policy decisions, and social norms. States are legal and political units that play a role in shaping environments that either inhibit or encourage structural racism through policy that shapes both racial inequality and reproductive healthcare access. Although not directly measured in our analysis, policies can shape individuals’ sexual health and behavior by limiting their access to care (Goyal et al., 2020; Redd et al., 2021), shaping their sexual education curriculum (Vivancos et al., 2013), and influencing whether individual’s feel comfortable using sexual health services (Gilmore & Somerville, 1994; Kirby et al., 2006; Lichtenstein et al., 2005), all of which have important implications for STI prevalence.

While our study sheds light on the relationship between structural racism and racialized STI patterns, our study is not without limitations. We are unable to investigate how state-level factors shape individual-level exposure to STIs. This is an important area for future research as state-level factors might interact with individual-level factors that shape sexual and reproductive health care access and use (Freitas Goes et al., 2021; Goyal et al., 2020; Newmyer & Frisco, 2023). Moreover, our analysis only investigates exposure to structural racism and its association with racialized disparities in STIs at the state level. It may be that different spatial scales are more appropriate for understanding the foundational relationship between structural racism and sexual health (Hing et al., 2024). For example, in the interconnected domains of housing, education, and the economy, residential segregation at the tract level in comparison to the county level or segregation within a municipality between the principal city, inner-ring suburbs, outlying suburbs and the suburban fringe could have varying implications for racialized dating opportunities, access to sexual health care, and education around sexual health (Lichter et al., 2015, 2023). The relationship between structural racism and STIs may also look different at the neighborhood, school, and interpersonal relationship level. Future analysis should consider applying multi-level analysis to investigate how macro-, meso-, and micro-level factors interact to shape racialized STI patterns.

Additionally, our analysis only investigates Black–White racialized disparities in STI patterns, but CDC estimates indicate that other racial and ethnically marginalized populations experience disproportionate risk of exposure and contraction of STIs in comparison to their White counterparts (Centers for Disease Control & Prevention, 2021). Further analyses are needed to theorize and analyze the structural mechanisms shaping disparate STI rates among other racial and ethnic groups. Though we replicate Brown and colleagues (2022) approach to measuring structural racism there may be certain domains of structural racism related to sexual health we do not capture in this measure. Moreover, we only capture contemporary exposure to structural racism which fails to consider how structural racism is embedded in historical racial regimes which continue to shape contemporary racial disparities (Baker, 2022). We encourage population health scholars to continue to work on identifying the multi-sectoral, systemic, and historically-embedded nature of structural racism (Hing et al., 2024; Hummer, 2023). Future research should also look more closely at policy changes related to reproductive healthcare and education programs regarding sexual health to investigate whether they shape racialized state-level STI rates.

Racialized disparities in STIs are consistent with the U.S.’s long documented history of the sexual and reproductive mistreatment of Black men and women, from the denial of treatment for syphilis among Black men in the Tuskegee Experiment to J. Marion Sims’ use of enslaved Black women’s bodies for gynecological medical experimentation without anesthesia (Roberts, 2016). Our study contributes to the literature aiming to shed light on the multi-sectoral, systemic, and hidden nature of structural racism and its role in explaining persistent racialized disparities in patterns of population health. However, our findings indicate that there continues to be unmeasured social, structural, or cultural phenomena that contribute to the geography of racialized disparities in STIs beyond structural racism. Further research is needed to better understand the multiple factors that shape racialized geographic patterns of STIs, as well as other sexual health outcomes.

## Appendix

See Tables 3, 4, 5, 6, 7, 8, 9, 10, and Fig. 4.

**Table 3:** Correlation Matrix of Covariates Included in Analysis

Panel A. Covariates Included in Main analysis and Appendix Table 4										
Black/White STI Rate Ratio	1.00									
Latent Structural Racism	.28	1.00								
Total Population, Logged	− .08	− .38	1.00							
Gini Index of Income Inequality, Standardized	− .09	− .19	.12	1.00						
Percent Black–White Interracial Marraiges	− .19	− .06	− .25	.48	1.00					
Voted for Republican Presidential Candidate	− .09	− .18	− .04	− .06	− .25	1.00				
Anti-Black Prejudice, Google Searches using N* Word	.09	− .11	− .17	.08	− .10	.58	1.00			
Percent Black	− .14	.13	− .18	.53	.55	.10	.31	1.00		
Percent in Poverty	− .14	− .15	− .05	.37	.00	.57	.67	.44	1.00	
Region	− .42	− .22	.01	− .06	.22	.18	.11	.17	.38	1.00

Panel B. Covariates Included in Latent Structural Racism Measure										
B/W Arrest Rate	1.00									
W/B College Completion Race	.27	1.00								
B/W Unemployment Rate	.22	.27	1.00							
B/W Poverty Rate	.44	.55	.38	1.00						
W/B Homeownership Rate	.26	.17	.07	.40	1.00					
W/B Voting Rate	.23	.20	.11	.33	.39	1.00				
White Isolation Index	.20	− .14	− .11	.00	.54	.36	1.00

**Table 4:** Random-Effects Spatial Autoregressive Models Assessing Potential Confounding Measures Predicting State-Level STI Rates per 100,000 between 2010 and 2020, N = 418

	Percent Black				Percent in Poverty				Region				Percent Black–White Interracial Marriages				Anti-Black Prejudice, Google Searches using N* Word			
	Model 1		Model 2		Model 1		Model 2		Model 1		Model 2		Model 1		Model 2		Model 1		Model 2	
	b	SE	b	SE	b	SE	b	SE	b	SE	b	SE	b	SE	b	SE	b	SE	b	SE
Latent Structural Racism	.63***	(.15)	.59***	(.16)	.65***	(.15)	.65***	(.15)	.56***	(.15)	.63***	(.15)	.66***	(.15)	.67***	(.15)	.66***	(.14)	.66***	(.15)
*State-Level Covariates*																				
Total Population, Logged			.072	(.31)			− .12	(.27)			− .042	(.27)			.16	(.28)			− .17	(.29)
Gini Index of Income Inequality, Standardized			− .37	(.32)			.3	(.29)			− .026	(.27)			− .067	(.23)			.18	(.25)
Percent Black–White Interracial Marraiges			− 3.8***	(.69)			− 3.3***	(.66)			− 3***	(.7)	− 3.2***	(.66)					− 3.3***	(.67)
Voted for Republican Presidential Candidate			− .092	(.24)			.055	(.25)			− .0049	(.24)			.12	(.24)			.061	(.24)
Anti-Black Prejudice, Google Searches using N* Word			.28*	(.13)			.36***	(.13)			.31*	(.13)			.31*	(.13)	.31*	(.13)		
*Other Cofounders*																				
Percent Black	.036	(.024)	.089**	(.033)																
Percent in Poverty					.025	(.079)	− .11	(.11)												
*Region (ref. Northeast)*																				
Midwest									− .74	(.71)	− 1.3	(.87)								
South									− 1.5*	(.64)	− 1.5*	(.72)								
West									− 2.8***	(.81)	− 2.5**	(.91)								
Spatial Error Variance Parameter	.58***	(.041)	.49***	(.061)	.58***	(.043)	.58***	(.058)	.58***	(.041)	.53***	(.053)	.53***	(.047)	.59***	(.045)	.59***	(.041)	.54***	(.05)
Constant	5.4***	(.43)	5.7	(4.9)	5.6***	(1.2)	11*	(4.7)	7.1***	(.56)	9.6*	(4.4)	7.8***	(.47)	3.4	(4.3)	5.9***	(.26)	10*	(4.6)
Pseudo R-Squared	0.074		0.136		0.070		0.148		0.221		0.213		0.099		0.090		0.091		0.104	
AIC	1436		1408		1438		1415		1430		1413		1413		1437		1432		1419	
BIC	1460		1452		1462		146+0		1463		1465		1438		1474		1456		1455	

Note: The Black/White STI Rate Ratio represent the racialized disparity in the incident rate of STIs for the population aged 44 and younger. The data covers the 37 continental states with a Black population of at least 50,000 and Washington D.C. This sample is consistent with studies of structural racism as it contains over 99% of the U.S. Black population. The Wald test of spatial autocorrelation is significant at p < .05. The spatial matrix for the spatial error term uses the Queen 1 criterion. *p < .05, **p < .01, ***p < .001

**Table 5:** Random-Effects Spatial Autoregressive Models Predicting Elder State Level Black/White STI Rate Ratio per 100,000 between 2010 and 2020, N = 418

	Model 1		Model 2	
	b	SE	b	SE
Latent Structural Racism	.79*	(.36)	.28	(.35)
*State-Level Covariates*				
Total Population, Logged			− .81	(.61)
Gini Index of Income Inequality, Standardized			− 2.9***	(.64)
Percent Black–White Interracial Marraiges			− 6***	(1.50)
Voted for Republican Presidential Candidate			− .94	(.58)
Antl-Black Prejudice, Google Searches using N* Word			.18	(.32)
Spatial Error Variance Parameter	.59***	(.04)	.47***	(.05)
Constant	8.7***	(.67)	25**	(9.7)
Pseudo R-Squared	0.046		0.363	
AIC	2182		2134	
BIC	2203		2174	

The Black/White STI Rate Ratio represent the racialized disparity in the incident rate of STIs for the population aged 45 and older. The data covers the 37 continental states with a Black population of at least 50,000 and Washington D.C. This sample is consistent with studies of structural racism as it contains over 99% of the U.S. Black population. The Wald test of spatial autocorrelation is significant at *p* < .05. The spatial matrix for the spatial error term uses the Queen 1 criterion. **p* < .05, ***p* < .01, ****p* < .001

**Table 6:** Random-Effects Spatial Autoregressive Models Assessing State-Level Individual Black/White STI Rate Ratios per 100,000 between 2010 and 2020, N = 418

	Chlamydia				Gonorrhea				Syphilis			
	Model 1		Model 2		Model 1		Model 2		Model 1		Model 2	
	b	SE	b	SE	b	SE	b	SE	b	SE	b	SE
Latent Structural Racism	.67***	(.17)	.70^***^	(.17)	1.5***	(.26)	1.3***	(.26)	.34	(.27)	.19	(.26)
*State-Level Covariates*												
Total Population, Logged			− .58	(.33)			.67	(.54)			.14	(.37)
Gini Index of Income Inequality, Standardized			.95***	(.27)			− 1.9***	(.46)			− 1.1***	(.31)
Percent Black–White Interracial Marraiges			− 4.4***	(.78)			− 1.9	(1.1)			− 1.7	(1.2)
Voted for Republican Presidential Candidate			− .18	(.27)			.82*	(.41)			.01	(.45)
Anti-Black Prejudice, Google Searches using N* Word			.35*	(.15)			− .12	(.22)			.35	(.27)
Spatial Error Variance Parameter	.56***	(.05)	.57***	(.05)	.72***	(.03)	.7***	(.03)	.25***	(.064)	.21**	(.07)
Constant	5.8***	(.28)	17***	(5.2)	8.3***	(.6)	− 1.3	(8.6)	5.1***	(.34)	3.9	(6)
Pseudo R-Squared	0.108		0.119		0.059		0.230		0.005		0.128	
AIC	1569		1533		1932		1911		2003		1990	
BIC	1589		1574		1952		1952		2023		2030	

The Black/White STI Rate Ratio represent the racialized disparity in the incident rate of STIs for the population aged 44 and younger. The data covers the 37 continental states with a Black population of at least 50,000 and Washington D.C. This sample is consistent with studies of structural racism as it contains over 99% of the U.S. Black population. The Wald test of spatial autocorrelation is significant at *p* < .05. The spatial matrix for the spatial error term uses the Queen 1 criterion. **p* < .05, ***p* < .01, ****p* < .001

**Table 7:** Random-Effects Spatial Autoregressive Models Assessing Alternative Latent Structural Racism Measure and State-Level Black/White STI Rate Ratio per 100,000 between 2010 and 2020, N = 418

	Model 1		Model 2	
	b	SE	b	SE
Alternative Latent Structural Racism	.64^***^	(.13)	.63***	(.13)
*State-Level Covariates*				
Total Population. Logged			− .12	(.28)
Gini Index of Income Inequality, Standardized			.08	(.24)
Percent Black–White Interracial Marraiges			− 3.30***	(.67)
Voted for Republican Presidential Candidate			− .09	(.24)
Anti-Black Prejudice, Google Searches using N* Word			.30*	(.13)
Spatial Error Variance Parameter	.56***	(.04)	.50***	(.05)
Constant	5.9***	(.25)	9.7*	(4.5)
Pseudo R-Squared	0.130		0.151	
AIC	1430		1410	
BIC	1450		1450	

The alternative latent structural racism measure incorporates both a tract and county level measure of the White isolation index of segregation. The Black/White STI Rate Ratio represent the racialized disparity in the incident rate of STIs for the population aged 44 and younger. The data covers the 37 continental states with a Black population of at least 50,000 and Washington D.C. This sample is consistent with studies of structural racism as it contains over *99%* of the U.S. Black population. The Wald test of spatial autocorrelation is significant at *p* < .05. The spatial matrix for the spatial error term uses the Queen 1 criterion. **p* < .05, ***p* < .01, ****p* < .001

**Table 8:** Random-Effects Spatial Autoregressive Models Assessing Alternative Latent Structural Racism Measure and State-Level Black/White STI Rate Ratio per 100,000 between 2010 and 2020, N = 418

	Brown et al.’s (2022) Latent Structural Racism Measure			
	Model 1		Model 2	
	b	SE	b	SE
Latent Structural Racism	.61***	(.14)	.60***	(.14)
*State-Level Covariates*				
Total Population, Logged			− .18	(.28)
Gini Index of Income Inequality, Standardized			.14	(.24)
Percent Black–White Interracial Marraiges			− 3.2***	(.67)
Voted for Republican Presidential Candidate			− .05	(.24)
Anti-Black Prejudice, Google Searches using N* Word			.32*	(.13)
Spatial Error Variance Parameter	.58***	(.04)	.53***	(.051)
Constant	5.9***	(.25)	10*	(4.5)
Pseudo R-Squared	0.120		0.138	
AJC	1437		1417	
BIC	I457		1457	

The latent structural racism presented here is an exact replicate of Brown et al.'s (2022). The Black/White STI Rate Ratio represent the racialized disparity in the Incident rate of STIs for the population aged 44 and younger. The data covers the 37 continental states with a Black population of at Least 50,000 and Washington D.C. This sample is consistent with studies of structural racism as it contains over 99% of the U.S. Black population. The Wald test of spatial autocorrelation is significant at *p* < .05. The spatial matrix for the spatial error term uses the Queen 1 criterion. **p* < .05, ***p* < .01, ****p* < .001

**Table 9:** Fixed-Effects Spatial Autoregressive Models Predicting Stale-Level Black/White STI Rate Ratio per 100,000 between 2010 and 2020, N = 418

	Model 1		Model 2	
	b	SE	b	SE
Latent Structural Racism	.72***	(.18)	.50**	(.19)
*State-Level Covariates*				
Total Population, Logged			− 13***	(3.5)
Gini Index of Income Inequality, Standardized			− 1.20**	(.43)
Percent Black–White Interracial Marraiges			− 4.10***	(.74)
Voted for Republican Presidential Candidate			.12	(.25)
Anti-Black Prejudice, Google Searches using N* Word			.37*	(.14)
Spatial Error Variance Parameter	.58***	(.04)	.30***	(.07)
Constant	1.1***	(.04)	1.l***	(.04)
Pseudo R-Squared	0.077		0.013	
AIC	1204		1154	
BIC	1217		1186	

The Black/White STI Rate Ratio represent the racialized disparity in the Incident rate of STIs for the population aged 44 and younger. The data covers the 37 continental states with a Black population of at Least 50,000 and Washington D.C. This sample is consistent with studies of structural racism as it contains over 99% of the U.S. Black population. The Wald test of spatial autocorrelation is significant at *p* < .05. The spatial matrix for the spatial error term uses the Queen 1 criterion. **p* < .05, ***p* < .01, ****p* < .001

**Table 10:** Random-Effects Spatial Autoregressive Models Assessing. Lags to Predict State-Level Black/White STI Pate Ratio per 100,000 between 2010 and 2020, N = 418

	Model 1		Model 2	
	b	SE	b	SE
Latent Structural Racism	.67***	(.14)	.66***	(.15)
*State-Level Covariates*				
Total Population, Logged			− .18	(.30)
Gini Index of Income Inequality, Standardized			.11	(.26)
Percent Black–White Interracial Marraiges			− 4.10***	(.69)
Voted for Republican Presidential Candidate			− .005	(.25)
Anti-Black Prejudice, Google Searches using N* Word			.17	(.13)
Spatial Error Variance Parameter	.59***	(.04)	.52***	(.06)
Constant	5.9***	(.26)	11*	(4.8)
Pseudo R-Squared	0.074		0.116	
AIC	1434		1405	
BIC	1454		1445	

All measures are lagged by 1 year, meaning that measures in 2010 predict die Black/White STI Rate Ratio in 2011 and so on. The Black/White STI Rate Ratio represent the racialized disparity in the incident rate of STIs for the population aged 44 and younger. The data covers the 37 continental states with a Black population of at least 50,000 and Washington D.C. This sample is consistent with studies of structural racism as it contains over 99% of the U.S. Black population. The Wald test of spatial autocorrelation is significant at *p* < .05. The spatial matrix for the spatial error term uses the Queen 1 criterion. **p* < .05, ***p* < .01, ****p* < .001

## Footnotes

1 The 13 excluded states include Alaska, Hawaii, Idaho, Maine, Montana, New Hampshire, New Mexico, North Dakota, Oregon, South Dakota, Utah, Vermont, and Wyoming.

2 Brown et al.’s (2022) original measure uses the Black–White dissimilarity index to represent segregation. We chose to use the White isolation index given increasing evidence that White neighborhood advantage is a primary driver of racialized inequalities (Howell, 2019). Given our results are robust when using either segregation measure to create the latent construct of structural racism, we present the main results using the White isolation index.
